# The Impact of the COVID-19 Pandemic on Medical Interns' Education, Training, and Mental Health: A Cross-Sectional Study

**DOI:** 10.7759/cureus.19250

**Published:** 2021-11-04

**Authors:** Nasser M AbuDujain, Qais A Almuhaideb, Nouf A Alrumaihi, Maha A Alrabiah, Mohammed H Alanazy, Hamza Abdulghani

**Affiliations:** 1 College of Medicine, King Saud University, Riyadh, SAU; 2 Department of Medical Education, College of Medicine, King Saud University, Riyadh, SAU; 3 Department of Internal Medicine, College of Medicine, King Saud University, Riyadh, SAU

**Keywords:** psychological impacts, mental health, medical interns, medical education, covid19

## Abstract

Background and objectives

The novel coronavirus disease 2019 (COVID-19) pandemic has challenged healthcare systems worldwide. Various studies have revealed the negative impact of the pandemic on the education and mental health of medical students and residents. In this study, we aimed to explore the effects of the COVID-19 pandemic on medical interns’ educational experience, clinical practice, and mental health. We also engage in a discussion on the compensatory methods that have been adopted to improve medical interns’ learning processes during the ongoing pandemic.

Methods

This cross-sectional survey-based study was conducted at the King Saud University Medical City (KSUMC) in Riyadh, Kingdom of Saudi Arabia (KSA) from March to October 2020. The participants consisted of medical interns. The survey collected information on participants’ demographics, training, and the educational and psychological impact of the pandemic.

Results

The survey was distributed to 480 medical interns, of whom 345 (71.8%) participated. Most of our sample (75.6%) believed that the COVID-19 pandemic has negatively impacted their cumulative experience. Regarding the pandemic’s impact on training and education, it was found that the emergency department rotation was the most affected rotation (60.3%). The majority of the participants (55.9%) believed that all tracks have received equal education and the volume of patients seen by interns decreased by 91.9%. As for the compensatory methods, 73.3% reported the utilization of remote platforms. Regarding the pandemic’s mental health impact, 36% believed that it has affected their mental health, where increased stress levels were noticed in 47.8% of participants. Statistical significance was found in both Patient Health Questionnaire-9 (PHQ-9) and General Anxiety Disorder-7 (GAD-7) scores, which indicated that during the pandemic, 20.6% of the participants suffered from depression, and 13.9% had moderate to severe anxiety.

Conclusions

This study highlights the negative educational and psychological impact of COVID-19 on medical interns. With emerging infectious diseases on the rise, recognizing the impact of COVID-19 on medical interns is vital to improving interns’ educational experiences and mental health during future crises.

## Introduction

On March 2, 2020, the first case of coronavirus disease 2019 (COVID-19) was reported in the Kingdom of Saudi Arabia (KSA) [1]. Even before confirming the first COVID-19 case in KSA, Saudi governmental authorities had introduced social distancing measures such as suspending mass gatherings and imposing a curfew [1]. By the end of March, the number of COVID-19 cases had reached approximately 1,600, and it jumped to 24,100 cases by the end of April [2]. The number of COVID-19 cases continued to rise, reaching a total of 300,000 confirmed cases with 24,942 active cases by the middle of August [3]. By September and October 2020, the number of recoveries started outweighing the positive cases, and the curve began to plateau [2].

This pandemic has wreaked havoc on a global scale by impacting many aspects of human life [4]. Many studies have explored the impact of the pandemic on various groups of healthcare workers (HCWs). One group within the healthcare system that has not received much attention in the literature is medical interns despite the significant impact of the pandemic on them. In KSA, the internship refers to the one-year mandatory supervised hospital training needed to obtain a bachelor’s degree in medicine and surgery [5].

Online educational methods have been considered to be an efficient tool for learning for a long time and are not really a novel concept in KSA. Due to the ongoing pandemic, an unplanned shift from traditional learning to online learning has occurred, causing medical institutions to change the delivery method of their courses. Despite the poor non-verbal communication associated with online learning, utilizing it in medical education can lead to easier and more effective access to information, especially during unprecedented worldwide events, such as pandemics [6].

Our literature review revealed a limited number of studies on the educational impact of COVID-19 on medical interns [7-9]. However, none of these studies has discussed the psychological impact of the pandemic on interns; moreover, national-level studies discussing the pandemic’s impact on interns have been scarce. In light of this, we conducted this study to explore the pandemic's effect on the educational and training experience as well as the mental health of medical interns. Additionally, we discuss the compensatory methods that were adopted to improve their learning process and help the interns cope with the current situation.

## Materials and methods

Study design and survey sample

This observational cross-sectional study was conducted at the King Saud University Medical City (KSUMC) and College of Medicine in Riyadh between March and October 2020. The total population sampling technique (non-probability sampling) was used to include all medical interns who had completed at least one rotation of their internship. The sample size was calculated to be 214 from a population of 480 based on a confidence level (CL) of 95% and a confidence interval (CI) of 5.

The questionnaire

An online self-administered open (non-closed) survey was developed via Google Forms. All medical interns were contacted directly through email or direct message and were invited to participate voluntarily, without offering any incentives. The survey comprised 35 items, distributed over five pages, and averaged nine items per page. No randomization of items or survey was done, and adaptive questioning was used in devolving the survey. The purpose of the study, the identity of the primary investigator, and the expected time to fill out the survey were disclosed. Participants were informed that they would be providing informed consent by clicking "next" on the page.

The survey collected information on interns' demographics, training, and educational and psychological impact of the pandemic. The training and education section included questions about clinical rotations, number of patients encountered, faculty engagement, research opportunities, overall cumulative experiences, and residency program matching, which is a system that uses a proprietary algorithm to match applicants to post-graduate training programs throughout accredited training centers. The training and educational section also included mitigation measures taken by the institute, which included COVID-19-related training and the utilization of remote platforms. The psychological impact section included questions on stress levels, prior diagnosis of mental illness before the pandemic, coping skills, support systems, and activities that improved mental health. The Patient Health Questionnaire-9 (PHQ-9) was employed, which is a nine-item scale used to diagnose depression and grade the severity of symptoms provisionally. The Generalized Anxiety Disorder-7 (GAD-7) questionnaire was also used, which is a seven-item scale used to screen for symptoms and measure the severity of generalized anxiety disorder. Both scales have similar scoring systems in which the score is calculated by allocating scores of 0, 1, 2, and 3 to the response categories of "not at all," "several days," "more than half the days," and "nearly every day," respectively. The cut-off point considered positive in our study was moderate depression and anxiety and above, which was equivalent to scoring 10 and above, and 11 and above for depression and anxiety, respectively [10]. No personal information was collected.

A completeness check was done by highlighting the mandatory items; some items provided a non-response option, and the selection of one response option was enforced. Participants were able to review and change their answers by using the back button. Duplicate entries would be formed if the participating intern clicked the refresh button after submitting the survey. Duplicate entries were avoided by manual deletion prior to analysis. Cookies and IP addresses were not used to identify potential duplicate entries, and no other techniques were used to identify multiple entries. No participation or view rates were provided; no timeframe was used as a cut-off point, and no statistical correction was done. 

By ensuring that Google Forms only submitted fully completed responses and by making at least one answer per item mandatory, a completion rate of 100% was achieved with no incomplete surveys. After the completion of responses, data were automatically transferred to a Microsoft Excel sheet. Data were stored on Google Drive, and it was only accessible to the research members throughout the research period.

Questionnaire validation

The survey was designed based on an extensive literature review and was validated by multiple revisions and editing. Moreover, additional validation was conducted via a pilot study consisting of 15 medical interns who reviewed and ensured item clarity and relatedness to their educational experience during the internship year. The survey’s reliability was tested and showed a Cronbach’s alpha level of 0.918 and 0.934 for PHQ-9 and GAD-7, respectively.

Ethical consideration

This study was conducted after obtaining approval from the King Saud University Institutional Review Board (Approval Reference No. 20/0817 | IRB. Date: 10/11/2020) and was performed according to the international code and standards of research ethics.

Statistical analysis

The data were analyzed using the SPSS Statistics software version 24 (IBM, Armonk, NY). Descriptive statistics [means, standard deviations (SDs), frequencies, and percentages] were used to describe the quantitative and categorical variables as appropriate. A p-value of <0.05 and a CI of 95% were used to report the statistical significance and precision of the results. No methods were used to adjust for a nonrepresentative sample.

## Results

In this study, 345 out of 480 interns filled out the survey, resulting in a response rate of 71.8%. As seen in Table 1, males constituted 49.6% of the participants. The participants' ages ranged from 22 to 28 years, with a mean ± SD of 24.51 ± 0.89 years; 4.9% of them were married. Most participants were King Saud University graduates (83.5%) and living in Riyadh (87.9%). Regarding the clinical rotations, the most common rotation that was affected by the COVID-19 pandemic was the emergency rotation as reported by 60.3% of the participants, followed by pediatrics (40.6%), internal medicine (40.3%), surgery (38.8%), elective (36.5%), and lastly, obstetrics and gynecology (24.9%).

**Table 1:** Medical interns’ demographic information and affected rotations OB/GYN: obstetrics and gynecology

Demographic information and affected rotations	
Variables	N (%)
Gender	
Male	171 (49.6)
Age in years	
22	1 (0.3)
23	26 (7.5)
24	164 (47.5)
25	116 (33.6)
26	28 (8.1)
27	8 (2.3)
28	2 (0.6)
Marital status	
Single	328 (95.1)
King Saud University graduate	
Yes	288 (83.5)
Riyadh resident	
Yes	303 (87.8)
Rotations affected by the pandemic	
Emergency	208 (60.3)
Medicine	139 (40.3)
Surgery	134 (38.8)
Pediatrics	140 (40.6)
OB/GYN	86 (24.9)
Elective	126 (36.5)

As shown in Table 2, the number of patients seen by interns during the pandemic had drastically diminished, as reported by 91.9% of the participants, while 7.5% believed that the patient volume had stayed about the same. Regarding the impact of COVID-19 on the interns’ training, 75.6% of the participants thought that the pandemic had a negative impact on their cumulative training experience, whereas 14.2% thought it had a positive effect. One aspect of the intern training was the measures taken by the departments during the pandemic to hinder the spread of the virus. Some of these measures reported by the interns included limits imposed on interns’ work/access (77.7%), admission/surgery restrictions (55.1%), duty subdivisions (54.2%), cancelation of rotations (46.4%), cancelation of case discussions and presentations (40.9%), and telemedicine usage (36.8%). With respect to the response to the question if they thought that these measures were suitable, the percentage of interns that agreed can be divided into several categories, as follows: (1) 55.7% on work limitation, (2) 49.6% on duty subdivision, (3) 41.7% on telemedicine usage, (4) 39.4% on admission/surgery restrictions, (5) 9.3% on cancelation of rotations, and (6) 17.1% on canceled case discussions and presentations.

**Table 2:** COVID-19 pandemic's impact on interns' training COVID-19: coronavirus disease 2019; OR: operating room; IM: internal medicine; OB/GYN: obstetrics and gynecology

Pandemic's impact on interns' training	
Variables	N (%)
The volume of patients seen each week in outpatient clinics, inpatient consultation during the COVID-19 pandemic	
Increased	2 (0.6)
Stayed about the same	26 (7.5)
Decreased	317 (91.9)
COVID-19 pandemic's impact on the cumulative experience of the internship training	
Positive impact	49 (14.2)
Unchanged	35 (10.1)
Negative impact	261 (75.6)
During the pandemic, do you believe that all tracks received equal guidance and education?	
Yes	193 (55.9)
If not, what was the cause?	
Due to rotations being affected or the rotation order	29 (8.4)
Due to the decreased hospital access (clinics/wards/OR)	16 (4.6)
Due to the different departments' approaches in guidance and education	4 (1.2)
From your own point of view, which of the measures taken by the department do you believe were suitable?	
No measures were suitable from my own point of view	30 (8.7)
Limits on interns’ access to the hospital or work responsibilities	192 (55.7)
Restrictions on elective admissions/surgeries	136 (39.4)
Subdivision of clinical duties among interns	171 (49.6)
Utilization of telemedicine	144 (41.7)
Cancelation of case discussions and presentations	59 (17.1)
Cancelation of rotations (IM, surgery, pediatrics, OB/GYN, other)	32 (9.3)
Taking extra or new responsibilities/training in managing COVID-19 patients	50 (14.5)

As displayed in Table 3, faculty engagement with interns during the pandemic compared to that before the pandemic was assessed; 58.5% of the sample believed that the level of faculty engagement decreased. Only 27.2% of participants reported receiving regular faculty check-ups and assessments on their educational needs. Around half of the participants (48.5%) believed that their research opportunities have decreased, while 12.5% believed that research opportunities have increased, and 44.3% had continued with previous research or started a new project. The impact of the pandemic on future residency program acceptance was assessed. Most of the participants mentioned that the pandemic would affect their chances of getting into the residency program of their choice either negatively (33.0%) or positively (9.3%), while 40.3% of the participants believed it would not affect their chances of acceptance at all.

**Table 3:** COVID-19 pandemic's impact on interns' education COVID-19: coronavirus disease 2019

Pandemic's impact on interns' education	
Variables	N (%)
Faculty engagement with the interns	
Greatly increased	14 (4.1)
Slightly increased	22 (6.4)
Stayed about the same	107 (31)
Slightly decreased	105 (30.4)
Greatly decreased	97 (28.1)
Regular check-ups by program faculty on educational needs	
Yes	94 (27.2)
No	251 (72.8)
Impact of COVID-19 on research opportunities	
Greatly increased	22 (6.4)
Slightly increased	21 (6.1)
Stayed about the same	135 (39.1)
Slightly decreased	64 (18.6)
Greatly decreased	103 (29.9)
Impact on conducting any new research or continuing old researches	
Yes	153 (44.3)
No	192 (55.7)
Impact of COVID-19 on chances to get into residency programs	
Yes, it will positively affect my chances of getting the residency program of my choice	32 (9.3)
Yes, it will negatively affect my chances of getting the residency program of my choice	114 (33)
No, it will not at all affect my chances of getting the residency program of my choice	139 (40.3)
I had been matched with my first choice of residency program before the pandemic had started	60 (17.4)

Table 4 shows the mitigation measures taken by KSUMC, which included the utilization of remote platforms and COVID-19-related interns’ training policies. Regarding remote platforms, 73.3% of the participants reported using them. As for COVID-19-related training measures, 63.2% of the participants received training on at least one of several categories: (1) infection control measures, (2) patient safety, (3) isolation, (4) breaking bad news, and (5) personal protective equipment (PPE). The training was provided at the highest rate for infection control measures and PPE, constituting 58.8% and 55.1% of those who received training, respectively.

**Table 4:** COVID-19 pandemic-related mitigation measures COVID-19: coronavirus disease 2019; PPE: personal protective equipment

COVID-19 pandemic-related mitigation measures	
Variables	N (%)
Utilization of remote platforms such as Zoom, GoToMeeting, Skype?	
No	92 (26.7)
Yes	253 (73.3)
Presentation	148 (42.9)
Lectures	156 (45.2)
Discussions	101 (29.3)
Rounds	65 (18.8)
Tests and evaluations	38 (11)
Level of satisfaction felt by medical interns in using remote platforms for educational purposes	
Generally positive	147 (42.6)
Indifferent	84 (24.3)
Generally negative	44 (12.8)
Received training on the medical care of COVID-19 patients from the hospital or the department of the rotation	
Yes	218 (63.2)
No	127 (36.8)
COVID-19 medical care training	
Infection control measures	203 (58.8)
PPE donning, using, doffing, and disposing	190 (55.1)
Isolation	102 (29.6)
Patient safety	82 (23.8)
Breaking bad news	12 (3.5)

As shown in Table 5, one-third of the participants (36%) believed that the pandemic had affected their mental health. The participants were asked about the pandemic’s impact on their stress levels and daily activities. The pandemic has caused an increase in the stress levels of 47.8% of the participants, while it did not affect 37.4% of the participants. On the contrary, 14.8% experienced an improvement in their stress levels. Work was the most predominant cause of the increase in stress (27.5%), followed by stress from home (15.9%), fear of infection (3.2%), financial stress (2.9%), and stress from residency matching (1.2%). PHQ-9 and GAD-7 scores were used to assess the presence of depression and anxiety, respectively. Some (20.6%) of the participants had high scores corresponding to moderate to severe depression (p<0.001) with a median score and interquartile range (IQR) of 4 (9), while 13.9% of the participants had moderate to severe anxiety (p<0.001) with a median score and IQR of 3 (7). During the pandemic, 41.2% received support, mainly informally, from family, friends, and/or colleagues.

**Table 5:** Interns mental health during the pandemic IQR: interquartile range

Mental health of interns during the pandemic					
Variables	Values				
Before the pandemic, had you struggled with any of the following psychological disorders? n (%)					
Anxiety	78 (22.6)				
Depression	44 (12.8)				
Other	5 (1.4)				
Do you feel that the COVID-19 pandemic has affected your mental health? n (%)					
Yes	125 (36.2)				
What is the effect of the COVID-19 pandemic on your stress levels? n (%)					
Increased	165 (47.8)				
Stayed about the same	129 (37.4)				
Decreased	51 (14.8)				
Depression and anxiety among medical interns during the COVID-19 pandemic n (%)					
No depression			193 (55.9)		
Mild depression			81 (23.5)		
Moderate to severe depression			71 (20.6)		
No anxiety			230 (66.7)		
Mild anxiety			67 (19.4)		
Moderate to severe anxiety			48 (13.9)		
Comparison of depression and anxiety among male and female medical interns					
		Males		Females	P-value
PHQ-9 score, median (IQR)		2 (7)		5 (7)	<0.001
Moderate to severe depression, n (%)		29 (17%)		42 (24.1%)	0.10
GAD7 score, median (IQR)		1 (5)		5 (7)	<0.001
Moderate to severe anxiety, n (%)		15 (8.8%)		33 (19%)	0.006

The pandemic affected the daily activities of the interns. As displayed in Table 6, 62.9% of the intern had more social interactions before the pandemic, whereas 20.3% had experienced no change, and 16.8% had a more active social life during the pandemic. The pandemic did not affect the studies of 44.3% of the participants, the exercise of 38.3%, and relaxation of 53%. Moreover, about half of the participants (56.5%) had the chance to practice their hobbies more actively during the pandemic, which was mentioned to be the most useful activity for improving mental health (61.2%). Other activities that contributed to improving mental health included exercise (38.8%), relaxation (28.7%), social life (24.6%), and studies (22%).

**Table 6:** COVID-19 pandemic's effect on interns’ activities COVID-19: coronavirus disease 2019

Pandemic's effect on interns’ activities				
	Practiced more before the pandemic, n (%)	Unchanged, n (%)	Practiced more during the pandemic, n (%)	Improvement of mental health during the pandemic due to the activity, n (%)
Hobbies (indoor games, watching TV series/movies, social media surfing, reading, swimming, etc.)	53 (15.4)	97 (28.1)	195 (56.5)	211 (61.2)
Studies (extracurricular or curricular)	105 (30.4)	153 (44.3)	87 (25.2)	76 (22)
Relaxation (meditation, yoga, etc.)	50 (14.5)	183 (53)	112 (32.5)	99 (28.7)
Exercise (sports, weightlifting, etc.)	98 (28.4)	132 (38.3)	115 (33.3)	134 (38.8)
Social life activities (family gatherings, meeting friends, etc.)	217 (62.9)	70 (20.3)	58 (16.8)	85 (24.6)

## Discussion

The study aimed to measure the effect of the COVID-19 pandemic on the training of medical interns at a tertiary university hospital and analyze the psychological impact at the time of the pandemic.

In response to the pandemic, hospitals have implemented several precautions to decrease the rate of spread of COVID-19. These measures include reducing elective admissions, cancelation of departmental educational activities, utilization of telemedicine in outpatient settings and clinical rounds, and modifying the working hours of the trainees to reduce unwarranted exposure to coronavirus in hospitals [11]. As previously mentioned, similar measures have been taken by KSUMC authorities as well.

The precautions taken concerning medical interns vary among healthcare organizations. Whereas some institutions chose to continue on the same schedule as before the outbreak, others postponed or shortened interns’ training, and some interns have had their rotations canceled or made optional [12,13]. About half (46.4%) of our participants had one or more canceled rotations. In this study, the most affected rotation was emergency, which had been canceled for 60.3% of interns. This finding can be explained by the fact that all patients who first present to the emergency department have an unknown status with respect to COVID-19, which may increase the risk of unnecessary exposure. In addition, the elective rotation was canceled for only 36.5% of our participants. In comparison, a study conducted in the United Kingdom among final-year medical students showed that 77.3% had their electives canceled, mostly due to travel restrictions [12]. A systematic review of the medical and surgical education challenges in the COVID-19 era revealed that the reduction of elective surgeries and the postponement of clinical clerkships and observerships will inevitably affect surgical and medical education [14]. This may explain why 23.5% of our participants believed that they did not receive an equal education compared to other interns who had different rotation orders.

Moreover, many additional measures have been implemented in KSUMC; for example, the internship was postponed for one month for the new interns at the start of the internship year. Some interns with certain medical conditions were at high risk for contracting the infection. Attending work was further postponed for high-risk interns until approved by the internship office. In addition, the emergency department rotation was declared optional for the high-risk interns.

A study assessing the COVID-19’s impact on post-graduate year 1 (PGY-1) physicians showed a generally unfavorable effect on medical training, as high as 77% taking a dim view in connection with the medical school community and 55% regarding preparation for the intern year [15]. We found that 75% of the study participants believed that COVID-19 had a negative impact on their overall educational experience. Similarly, a study conducted in India showed that 70% of medical and surgical residents felt that the pandemic had resulted in a negative clinical impact [16].

Similarly, a study done in Makkah showed that 70.4% of medical interns reported that the pandemic affected their training and achievement of clinical skills [17]. On the other hand, a smaller percentage of our participants believed that the pandemic positively impacted their experience, which could be attributed to the decrease in workload [13].

A cross-sectional study in the United Kingdom involving medical students in their assistantship year showed that 93.9% believed that the measures taken during the pandemic were necessary [12]. Similarly, in our study, 91.3% believed that the measures taken were suitable.

A systematic review of the immediate consequences and solutions applied to maintain medical education during the COVID-19 pandemic for residents and medical students revealed that the most frequently attempted solution was utilizing online courses [18]. Most of the analyzed studies reported an accelerated adaptation of videoconferencing and remote clinical sessions [18]. In our study, 72% of the interns used remote platforms during their rotations, primarily for lectures and presentations. One study has shown that virtual learning can be useful during the COVID-19 lockdown; 75.7% of the respondents felt that online classes and webinars were useful during this lockdown period [19]. A study done among medical interns in Makkah showed similar results, where 55.5% of medical interns felt that remote education was sufficient for their pre-graduation needs [17]. Moreover, in the earlier systematic review, several studies showed a high level of satisfaction with remote lectures, virtual cases, and remote standardized patient encounters [8,9,18,20]. Similarly, in our research, the majority (42.6%) of the participants reported their satisfaction regarding remote platform utilization. This is in line with the findings of another study, in which 65.6% of interns considered remote classes to be of equal or better quality than in-person classes [21]. Regarding remote platforms utilization, 73.3% of our participants reported utilizing remote platforms at least once during the pandemic.

Various studies have reported on the significant impact of COVID-19 on mental health. Studies have shown that HCWs caring for patients with COVID-19 reported high rates of anxiety, depression, and stress [22,23]. However, only minor attempts have been made to study the psychological impact associated with COVID-19 on medical interns. The internship is challenging even without a pandemic; new physicians face long working days, sleep disturbances, major emotional upheavals, and loss of autonomy. Multiple cross-sectional studies have assessed the prevalence of depressive symptoms among medical interns. Most of the studies have shown that interns had higher rates of depression compared to the general population [19]. A prospective cohort study with 740 interns showed that 25.7% of interns met the PHQ-9 criteria for depression during the internship [24]. In our study, rates of depression were similar; 20.6% of the participants had depression during the pandemic with a median score (IQR) of 4 (9) [11]. The presence of stress among medical interns has been reported to have a prevalence ranging from 11 to 40% in the literature. A study conducted among interns of King Khalid, King Abdulaziz, and King Fahd University Hospitals in KSA showed that nearly 73.0% of interns were under stressful conditions [5]. A systematic review of the prevalence of anxiety in medical students during the COVID-19 pandemic estimated the prevalence to be 28% [25]. A lower prevalence was seen in our study, in which 13.9% of the interns had moderate to severe anxiety with a median score (IQR) of 3 (7). Moreover, we found that females were 2.43 times more likely to have anxiety with a 95% CI of 1.27-4.67 (Table 5). Similarly, a study conducted during the Middle East Respiratory Syndrome-Coronavirus (MERS-CoV) outbreak in KSA showed that female participants were found to have more anxiety and fear of becoming infected [26].

Daily life activities of interns, such as practicing their hobbies, relaxation, social life, and studies, have improved during the pandemic and this contributed to improving their mental health. When the interns were asked about social support, only 41.2% mentioned receiving it, which mainly came informally from family, friends, and/or colleagues. A systematic review on the mental health impact of COVID-19 on HCWs has shown that the most frequently reported protective factor associated with a reduced risk of mental health problems was having sufficient social support. Moreover, the authors found that the preference for the type of psychological resources was related to the degree of psychological distress. HCWs with moderate and severe psychological distress more often preferred to receive care from psychiatrists or psychologists. On the other hand, HCWs with subthreshold and mild psychological distress preferred to seek care from family or relatives [27].

Strengths and limitations

One of the strengths of our study was that we contacted every intern individually, which yielded a high response rate. One of the limitations was the absence of a validated survey framework to assess the pandemic's educational impact. Moreover, the limited number of respondents (345) means that our findings cannot be generalized. Further multi-center studies with a higher number of participants are required to validate our findings.

## Conclusions

Based on our findings, most of the participating interns believed that the COVID-19 pandemic has negatively impacted their cumulative internship experience, and around one-third thought that the pandemic has affected their mental health. We recommend utilizing remote platforms and more frequent faculty engagement to ensure the continuity of high-quality medical education. We also recommend offering regular check-ups and assessments to ensure the interns’ mental wellbeing.
